# Correction to: Ethnic and minority group differences in engagement with COVID-19 vaccination programmes – *at Pandemic Pace; when vaccine confidence in mass rollout meets local vaccine hesitancy*

**DOI:** 10.1186/s13584-021-00470-0

**Published:** 2021-10-28

**Authors:** John A. Reid, Mzwandile A. Mabhala

**Affiliations:** Faculty of Health and Social Care, University of Chester, Riverside Campus, Castle Drive, Chester, CH1 1SL UK

**Correction to: Isr J Health Policy Res 10, 33 (2021)**

**https://doi.org/10.1186/s13584-021-00467-9**

In the original publication of this article [1] an error was introduced during the publication process in the reference numbering. This caused all citations after 13 to be incorrect. The citations in the original article have now been corrected.

## References

[1] Reid JA, Mabhala MA (2021). Ethnic and minority group differences in engagement with COVID-19 vaccination programmes – *at Pandemic Pace; when vaccine confidence in mass rollout meets local vaccine hesitancy*. Isr J Health Policy Res.

